# Nrf2 Deficiency Exacerbated CLP-Induced Pulmonary Injury and Inflammation through Autophagy- and NF-κB/PPARγ-Mediated Macrophage Polarization

**DOI:** 10.3390/cells11233927

**Published:** 2022-12-04

**Authors:** Jing Luo, Jin Wang, Jing Zhang, Aming Sang, Xujun Ye, Zhenshun Cheng, Xinyi Li

**Affiliations:** 1 Department of Anesthesiology, Zhongnan Hospital of Wuhan University, Wuhan 430071, China; 2 Department of Geriatrics, Zhongnan Hospital of Wuhan University, Wuhan 430071, China; 3 Department of Respiratory and Critical Care Medicine, Zhongnan Hospital of Wuhan University, Wuhan 430071, China; 4 Wuhan Research Center for Infectious Diseases and Cancer, Chinese Academy of Medical Sciences, Wuhan 430071, China; 5 Hubei Engineering Center for Infectious Disease Prevention, Control and Treatment, Wuhan 430071, China

**Keywords:** Nrf2, acute lung injury, pulmonary inflammation, autophagy, macrophage polarization

## Abstract

The balance between M1 and M2 macrophage polarization is involved in the regulation of pulmonary inflammation. Nuclear factor erythroid-derived 2-like 2 (Nfe2l2, also known as Nrf2), a nuclear transcription factor, is reported to play protective roles in acute lung injury (ALI) and inflammation, and increasing evidence indicates that the protective effects of Nrf2 are closely related to autophagy. This study aimed to explore whether Nrf2 is involved in sepsis-induced acute pulmonary injury and inflammation and in the role of macrophage polarization in the process. In the present study, sepsis patients, an Nrf2 knockout mouse that underwent cecal ligation and puncture (CLP), and lipopolysaccharide (LPS)-treated macrophage cell lines were employed to investigate the potential functions of Nrf2 in sepsis-induced lung injury and the underlying mechanisms. Clinical studies showed that the NRF2 mRNA level was inversely correlated with pulmonary inflammation and disease severity in patients with sepsis. Analyses in a CLP-treated Nrf2 knockout mouse model indicated that an Nrf2 deficiency promoted a CLP-induced increase in M1 macrophage polarization and apoptosis and inhibited CLP-induced upregulation of the autophagy level in lung tissues. Experiments in RAW264.7 cells revealed that Nrf2 overexpression inhibited M1 macrophage polarization but promoted M2 macrophage polarization by improving the autophagy, and Nrf2 overexpression promoted PPARγ but inhibited NF-κB nuclear translocation. In conclusion, these results indicate that Nrf2 plays a protective role in sepsis-induced pulmonary injury and inflammation through the regulation of autophagy- and NF-κB/PPARγ-mediated macrophage polarization.

## 1. Introduction

Sepsis is a life-threatening syndrome characterized by the maladjustment of the host response to invading pathogens, which leads to a series of severe organ dysfunction [1]. The lung is the foremost vulnerable organ during sepsis, and nearly 50% of patients with severe sepsis will develop acute lung injury (ALI) or even acute respiratory distress syndrome (ARDS), which is a leading cause of death from sepsis [2,3]. More and more evidence shows that the excessive secretion of inflammatory factors is involved in the development of ALI/ARDS, and the degree and duration of the inflammatory reaction ultimately determine the prognosis of patients with sepsis-related ALI/ARDS [4].

During lipopolysaccharide (LPS)-induced ALI development, the Nrf2 pathway is activated and exerts antioxidant and anti-inflammatory effects [5,6]. Nrf2 is a nuclear transcription factor containing a leucine zipper structure [7]. Under physiological conditions, Nrf2 binds to Keap1 and is ubiquitinated and degraded by the Keap1-Cul3-E3 ubiquitin ligase complex. Under cellular stresses, Nrf2 is separated from Keap1 and activated, translocating into the nucleus and inducing the expressions of anti-oxidant, anti-inflammatory, and pro-survival genes [8]. AMPK/Nrf2 signaling and its downstream HO-1 were reported to exert anti-inflammatory effects and promote the polarization of macrophages toward the M2 phenotype [9,10]. Using siRNA-mediated Nrf2 knockdown in an alveolar macrophage mouse cell line (MH-S), researchers found that Nrf2 played an anti-inflammatory role by promoting the polarization of M2 macrophages and inhibiting M1 macrophage polarization [11]. In addition, the regulation of macrophage polarization by dehydrocostus lactone, an anti-inflammatory substance, was partly achieved through the Nrf2 signal [12]. These results indicate that the protective role of Nrf2 regarding pulmonary inflammation is associated with macrophage polarization.

In 2010, researchers discovered an interaction between p62/sequestosome1 (SQSTM1, an autophagy adaptor) and Keap1 (a Nrf2 substrate adaptor for the Cul3 E3 ubiquitin ligase), thus revealing the relationship between the Nrf2-Keap1-ARE axis and autophagy [13,14,15,16,17]. There exist canonical and noncanonical regulatory pathways for Nrf2 signaling. In the canonical pathway, Nrf2 binds to Keap1, leading to its ubiquitination and proteasome degradation. In the noncanonical pathway, impaired autophagic flux leads to the accumulation of p62, which sequesters Keap1 such that it can no longer bind to Nrf2, leading to increased Nrf2 signaling [18]. In the study of intervertebral disc (IVD) degeneration, researchers found that Nrf2 expression was decreased in human nucleus pulposus cells, and Nrf2 deficiency induced the downregulation of autophagy-related genes, which promoted IVD degeneration [19]. It was reported that Nrf2 activation by tert-butyl hydroquinone provided protection effects in diabetic mice through the upregulation of autophagic proteins and autophagic flux [20]. In a study of intestinal ischemia/reperfusion, Nrf2 was reported to counteract pulmonary inflammatory responses by inhibiting autophagy [21]. These results indicate that the protective roles of Nrf2 are closely related to autophagy in different environments.

It is unclear whether Nrf2 is involved in cecal ligation and puncture (CLP)-induced pulmonary injury and inflammation, as well as the roles of autophagy and macrophage polarization in the process. In the present study, we first explored NRF2 gene expression in patients with sepsis, then used a CLP-treated mouse model and LPS-treated macrophage cell lines to determine the roles of Nrf2 in sepsis-induced pulmonary injury and inflammation, and further explored the underlying molecular mechanisms.

## 2. Materials and Methods

### 2.1. Clinical Study on Patients with Sepsis

Thirty patients with acute to recovery sepsis (age > 18 years) were recruited from the Department of Respiratory and Critical Care Medicine at Zhongnan Hospital of Wuhan University. Patients were diagnosed with sepsis based on the results of blood culture and organ dysfunction according to the SEPSIS-3 definitions [1,22], and those who had malignant tumors were excluded from the study. Thirty healthy volunteers were recruited as controls. Clinical and biological data of the subjects were collected and analyzed in Table 1. Peripheral blood samples were collected for mRNA extraction and NRF2 level analysis. Bronchoalveolar lavage fluids (BALFs) were collected for inflammatory factor measurement by ELISA. The severity of sepsis in patients at different stages was evaluated by the APACHE II score (range 0–71) [23]. The study gained approvement from the ethics committee of Zhongnan Hospital of Wuhan University, and all the subjects signed informed consent before the study began (ethics batch number: 2020105).

### 2.2. Sepsis-Induced CLP Model

Eight-week-old wild-type (WT) and Nrf2-knockout (Nrf2^−/−^) littermate male mice on a C57BL/6N background (purchased from Cyagen, Suzhou, China) were used for animal experiments. In all the animal experiments, unless otherwise specified, each group contained six mice. All animals were starved for 24 h and had free access to water before the experiments began. To construct an animal model of sepsis, cecal ligation and puncture surgery was performed as previously described [24]. Briefly, mice were anesthetized by intraperitoneal injection of pentobarbital sodium at a dose of 50 mg/kg, and then a 2 cm incision in the abdominal wall was made, through which the distal ileocecal valve was separated and 1/3 of the cecum was ligated with a 3-0 silk thread. The ligation end was punctured with an 18-gauge needle, a little stool was extruded, and then the peritoneum was closed by simple running sutures and the skin by interrupted sutures. The sham operation group was performed in the same procedure without cecal ligation and puncture. Immediately after the surgery, 50 mL/kg saline was injected subcutaneously for resuscitation. All the experiments were performed according to the Laboratory Guidelines for Animal Use and Care and gained approval from the Animal Biosafety Level 3 Laboratory of Wuhan University (No. ZN2021185).

### 2.3. Collection of Mouse Lung Tissues and BALFs

Pulmonary tissues were gently lavaged three times to collect BALFs, and 1 mL HBSS (Invitrogen, Carlsbad, CA, USA) was used for each lavage. The BALFs were centrifuged at 250× *g* for 10 min at 4 °C, and cell precipitation was resuspended with PBS for subsequent experiments. Lung tissues were divided into several parts: one part was used for mRNA and protein expression analyses, and other parts were used for histological and immunochemical/immunofluorescence analyses.

### 2.4. Histologic Analysis

Lung tissues were fixed with 4% paraformaldehyde, paraffin-embedded tissue sections were prepared, and hematoxylin-eosin (HE) staining was performed following the standard procedures. Lung injury index was calculated using the method as previously described [25]. Briefly, lung injury was scored based on the degrees of edema, hemorrhage, inflammatory cell infiltration, and histopathological changes, with 0: absent of injury; 1: modest injury; 2: intermediate injury; 3: widespread injury; and 4: severe injury.

### 2.5. RNA-Seq Analysis

Total RNA was extracted from the lung tissues of CLP-treated WT and Nrf2 knockout mice, using a RNeasy Mini Kit (Qiagen, Hilden, Germany) and following the standard procedures. RNA purity was detected by the Agilent 2100 bioanalyzer (Thermo Fisher Scientific, Waltham, MA, USA) to make sure the A260/280 ratio is in a range of 1.8–2.0 and the rRNA ratio is >1.0. Sequencing libraries were developed using the MGIEasy rRNA Depletion Kit (MGI) and MGIEasy RNA Directional Library Prep Set Kit (MGI), in accordance with the instructions of the manufacturer. After DNA was removed, Poly (A) RNAs were purified with a Dynabeads mRNA Purification Kit (Invitrogen, Carlsbad, CA, USA) and subjected to double-stranded complementary DNA (cDNA) synthesis. Then, cDNA fragments were blunted and ligated to sequencing adaptors. RNA sequencing was performed on the MGISEQ-2000 System (BGI, Wuhan, China), and data were analyzed using R software (v 3.6.3, R Foundation for Statistical Computing, Vienna, Astria). Differentially expressed mRNAs were screened according to a log2 fold change > 1and Q value < 0.05.

### 2.6. Immunofluorescence

Apoptotic cells were detected using an in situ cell death detection kit (Roche, Basel, Switzerland, 11684817910), in accordance with the instructions of the manufacturer. For immunofluorescence staining, samples were fixed in 4% paraformaldehyde for 1 h, washed completely with PBST (1xPBS added with 0.1% Triton X-100) for three times, and permeabilized with 0.1% Triton X-100 for 1 h at room temperature. Then, samples were incubated with primary antibodies including anti-iNOS (1:100, Invitrogen, Carlsbad, CA, USA, 14-5920-82), anti-CD206 (1:200, Invitrogen, Carlsbad, CA, USA, MA5-16871), anti-F4/80 (1:200, Invitrogen, Carlsbad, CA, USA, 41-4801-82), anti-LC3 (1:200, Affinity, Changzhou, China, AF5402), anti-LAMP2 (1:500, Boster, Wuhan, China, M01573-1), anti-NF-κB (1:200, Affinity, Changzhou, China, AF5006), and anti-PPARγ (1:200, Affinity, Changzhou, China, AF6284) for 10 h at 4 °C. After three times of washing with PBST, samples were incubated with secondary antibodies including rabbit anti-mouse IgG Ab (green-fluorescent Alexa Fluor 488, Invitrogen, Carlsbad, CA, USA, A-11059, 1 μg/mL in PBS), goat anti-rabbit IgG Ab (red-fluorescent Alexa Fluor 594, Invitrogen, Carlsbad, CA, USA, A-11012, 2 μg/mL in PBS), or DAPI at room temperature for 45 min. After washing again, samples were imaged with a fluorescence microscope or a confocal microscope (Zeiss LSM 710, Oberkochen, Germany).

### 2.7. Macrophage Isolation and Culture

Bone marrow-derived macrophages (BMDMs) were isolated as previously described [26]. Specifically, femur and tibia were isolated from tissues, and bone marrow cells were collected with Dulbecco’s Modified Eagle’s Medium (DMEM, Gibco, Thermo Fisher Scientific, Waltham, MA, USA). Then, samples were filtered with a 70 μm diameter cell strainer and centrifuged at 2000 rpm for 5 min at 4 °C. After supernatant was removed, the precipitate was resuspended in lysis buffer for red blood cell lysis. After centrifugation at 2000 rpm for 5 min at 4 °C again, the precipitate was collected and resuspended in DMEM supplemented with 10% FBS, 10 ng/mL recombinant macrophage colony-stimulating factor (Peprotech, Cranbury, NJ, USA, 315-02), and 1% penicillin/streptomycin. Cells were cultured at 37 °C and 5% CO_2_ for one week to make sure that they were completely differentiated into BMDMs. Culture media were changed every two days during the process. The purity of the cultured macrophages was detected by flow cytometry.

### 2.8. Transmission Electron Microscopy

The lung tissues were fixed in 2.5% glutaraldehyde (Sigma-Aldrich, Saint Louis, MO, USA, G5882) overnight at 4 °C. After washing, the tissues were fixed in 1% phosphate-buffered osmium tetroxide (Sigma-Aldrich, Saint Louis, MO, USA, O5500) for 1 h, then dehydrated in ethanol solutions, and embedded in Epon. Ultrathin sections were stained using alcoholic uranyl acetate (Polysciences, Warrington, PA, USA, 6159-44-0) and alkaline lead citrate (Sigma-Aldrich, Saint Louis, MO, USA, 15326) and viewed via a JEM 1230 transmission electron microscope (JEOL Ltd., Tokyo, Japan).

### 2.9. Flow Cytometry

The proportion of M1 macrophages was detected by flow cytometry. Cells were stained with PE-conjugated anti-mouse F4/80 (Invitrogen, Carlsbad, CA, USA, 50-4801-82) and FITC-conjugated anti-mouse CD86 (Invitrogen, Carlsbad, CA, USA, 11-0862-82) as the primary antibodies. Samples were detected with a FACSCanto Flow Cytometer (BD Biosciences, New York, NJ, USA), and FlowJo software (v 10.8, BD Biosciences, New York, NJ, USA) was used for data analyses.

### 2.10. Induction of M1/M2 Polarization

LPS and IFN-γ (concentrations of 15 ng/mL and 50 ng/mL, respectively, Sigma-Aldrich, Saint Louis, MO, USA, L2880-10MG; Abbkine, Wuhan, China, PRP1015) were added in the culture media for M1 macrophage polarization. IL-4 and IL-13 (concentrations of 25 mg/mL, Abbkine, Wuhan, China, PRP2117; Abbkine, Wuhan, China, PRP100398) were added in the culture media for M2 macrophage polarization. Cells were induced and cultured for 24 h and collected for further experiments.

### 2.11. Cell Culture and Plasmid Transfection

The RAW264.7 cell line was purchased from the American Type Culture Collection (ATCC), cultured at 37 °C and 5% CO_2_, and DMEM-supplemented with 10% FBS, and 1% penicillin (Sigma-Aldrich, Saint Louis, MO, USA, A0166) served as cell culture medium. Transfection of Nrf2 plasmid was performed referring to the working instructions, and cells transfected with an empty vector were used as controls. After 48 h of culture, cells were collected for further experiments. In cell experiments, at least three replicates were performed for samples in each group.

### 2.12. Drug Administration

For macrophage polarization detection, BMDMs from both WT and Nrf2^−/−^ mice were divided into control group and LPS-treated group. In each group, BMDMs were incubated with DMSO or LPS (1 μg/mL) for 6 h. The cells were then collected for flow cytometry analyses or immunofluorescence staining. For autophagy activation or inhibition, 0.1 μM Rapamycin (RAPA, LC Laboratories, Woburn, MA, USA, AY22989) or 2 mM 3-MA (Sigma-Aldrich, Saint Louis, MO, USA, M9281) were added in the culture media with the macrophage polarization inducers simultaneously. After 24 h of culture, cells were collected for subsequent analyses. The recombinant mRFP-GFP-LC3B lentivirus vector was purchased from Hanbio Biotechnology (Shanghai, China, HB-AP2100001). Cells were transfected with mRFP-GFP-LC3B plasmid after drug administration and harvested for autophagy analyses 24 h later.

### 2.13. Quantitative Real-Time PCR

Total RNA was extracted using Trizol reagent (Invitrogen, Carlsbad, CA, USA). RevertAid First Strand cDNA Synthesis Kits (Thermo Fisher Scientific, Waltham, MA, USA) were used for cDNA synthesis. The qRT-PCR was performed on a CFX-96 real-time PCR detection machine (Bio-Rad, Hercules, CA, USA). The relative expression levels of the target genes were standardized to β-actin and calculated by using the 2^−ΔΔCT^ method. All the experiments were performed in triplicate.

### 2.14. Western Blot

Tissues or cell suspensions were collected for lysis and protein extraction, and the protein concentration was determined by a BCA protein assay kit (Abcam, Shanghai, China). Samples were electrophoresed in 8–12% SDS-PAGE gels, transferred to PVDF membranes (Millipore, Hayward, CA, USA), and then incubated with primary antibodies including rabbit anti-Nrf2 (1:1000, Affinity, Changzhou, China, AF7904), mouse anti-β actin (1:10,000, Affinity, Changzhou, China, T0022), rabbit anti-Bcl-2 (1:1000, Affinity, Changzhou, China, AF6139), rabbit anti-Bax (1:1000, Affinity, Changzhou, China, AF0120), rabbit anti-Cleaved-caspase3 (1:1000, Affinity, Changzhou, China, AF7022), rabbit anti-LC3 (1:1000, Affinity, Changzhou, China, AF5402), rabbit anti-Beclin1 (1:1000, Affinity, Changzhou, China, AF5128), rabbit anti-p62 (1:1000, Affinity, Changzhou, China, AF5384), rabbit anti-NF-κB (1:1000, Affinity, Changzhou, China, AF5006), rabbit anti-PPARγ (1:1000, Affinity, Changzhou, China, AF6284), and rabbit anti-Lamin B (1:10,000, proteintech, Wuhan, China, 12987-1-AP) at 4 °C overnight and HRP-conjugated secondary antibody (proteintech, Wuhan, China, 1:5000) at room temperature for 1 h. Target bands were detected using an enhanced chemiluminescence system, and quantitative analyses were performed using the Image J software (v 1.48, National Institutes of Health, NIH, USA).

### 2.15. Statistical Analysis

Continuous variables were represented as mean ± standard deviation (mean ± SD) or median (interquartile range) based on their distributions, and analyzed by Student’s *t*-test or Mann–Whitney *U* test. Categorical variables were analyzed by chi-squared test. For three or more groups, data differences were analyzed by one-way ANOVA. The significance of correlations was evaluated by linear correlation analysis using Pearson’s test. All the statistical analyses were performed with SPSS 20.0 (SPSS Inc., Chicago, IL, USA) and GraphPad Prism 5.0 (San Diego, CA, USA), and a two-tailed *p* value less than 0.05 was considered statistically significant.

## 3. Results

### 3.1. In Sepsis Patients, the NRF2 mRNA Level in Peripheral Blood Is Inversely Correlated with Inflammation and Disease Severity

**Figure 1:**
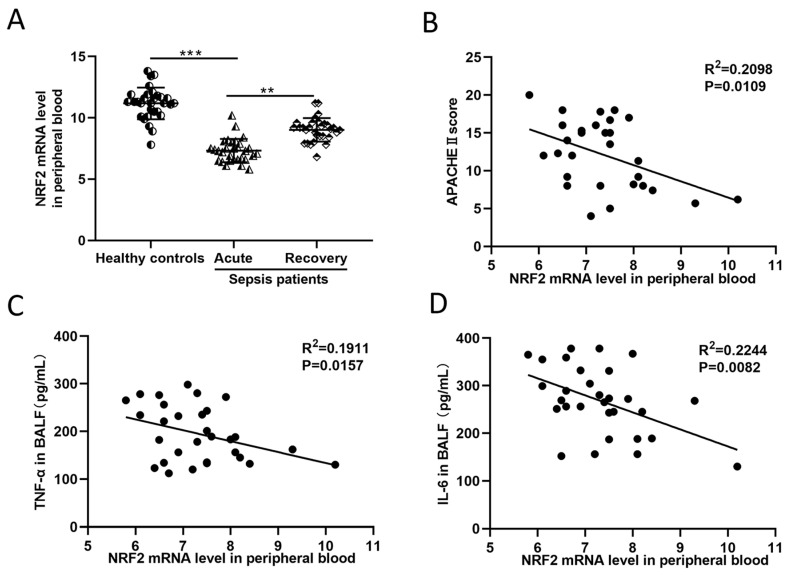
Patients with sepsis show a negative correlation between NRF2 mRNA level in peripheral blood and the severity of disease. (**A**) QRT-PCR detected the NRF2 mRNA level in the blood of 30 healthy controls and 30 sepsis patients at the acute and recovery stages. Paired *t*-test was used for data comparison between acute and recovery sepsis patients, and the NRF2 mRNA level was standardized to human housekeeping gene ACTB. (**B**) Correlation analysis of the APACHE II score and NRF2 mRNA level in the blood of acute sepsis patients. (**C**,**D**) Correlation analysis of TNF-α and IL-6 contents in BALFs and NRF2 mRNA level in the blood of acute sepsis patients. ** *p* < 0.01, *** *p* < 0.001.

Thirty patients with sepsis and thirty healthy volunteers were enrolled in the study. Table 1 listed the demographic data, comorbidities, and laboratory results of the healthy controls and sepsis patients. It can be concluded from Table 1 that in acute sepsis patients, the amounts of WBC and NEU were increased, and the expressions of inflammatory indicators such as PCT and CRP were upregulated as well. Blood gas analysis showed that sepsis patients had lower levels of SaO_2_, PO_2_, PH, and BE but higher levels of PCO_2_ and Lac. The BNP level was also upregulated in sepsis patients (*p* < 0.05 for all). For NRF2 mRNA measurement, peripheral blood samples from sepsis patients during acute illness and recovery stage were collected. The NRF2 mRNA level in the blood of healthy controls was notably higher than that in sepsis patients (Figure 1A). Correlation analysis revealed that the NRF2 mRNA level in the blood of sepsis patients was negatively related to their APACHE II scores, an indicator used for the assessment of disease severity in critical patients (Figure 1B). In addition, the blood NRF2 mRNA level was also negatively related to the inflammatory factor concentrations in the BALFs of sepsis patients (Figure 1C,D). These results suggested that in patients with sepsis, the NRF2 mRNA level in peripheral blood was inversely correlated with disease severity.

### 3.2. Nrf2 Deficiency Exacerbates Sepsis-Induced ALI and Promotes Inflammation in a CLP Mouse Model

**Figure 2:**
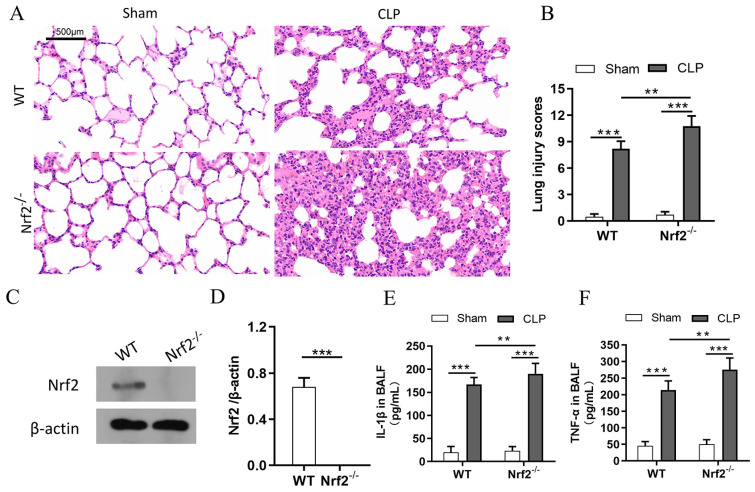
Nrf2 deficiency exacerbates sepsis-induced ALI and promotes inflammation in a CLP mouse model; n = 6 mice per group. (**A**) Histological analyses of the lung tissues in WT and Nrf2^−/−^ mice in both the sham and CLP groups. (**B**) Representative lung injury scores in (**A**). (**C**,**D**) Western blot analyses of Nrf2 protein in WT and Nrf2^−/−^ mice. (**E**,**F**) The expressions of inflammatory cytokines including IL-1β and TNF-α in BALFs were measured with ELISA. ** *p* < 0.01, *** *p* < 0.001.

In the present study, a mouse model with CLP-induced ALI was established. Compared with the sham group, the CLP group showed obvious pulmonary injuries such as edema, hemorrhage, and inflammatory cell infiltration. To analyze the role of Nrf2 in lung injury and inflammation, WT and Nrf2^−/−^ mice were subjected to CLP, and the HE staining results showed that Nrf2^−/−^ mice had higher lung injury scores compared with WT mice (Figure 2A,B). Western blot results showed that Nrf2 protein level was significantly decreased in Nrf2^−/−^ mice (Figure 2C,D). To further explore the pulmonary inflammatory response after Nrf2 knockout, the expressions of inflammatory cytokines such as IL-1β and TNF-α in BALFs were measured. As shown in Figure 2E,F, Nrf2 deficiency significantly enhanced the CLP-induced upregulation of IL-1β and TNF-α in BALFs, suggesting that Nrf2 protects lung tissues from CLP-induced inflammation.

### 3.3. Transcriptome Sequencing Analyses of Lung Tissues in CLP-Treated WT and Nrf2^−/−^ Mice

**Figure 3:**
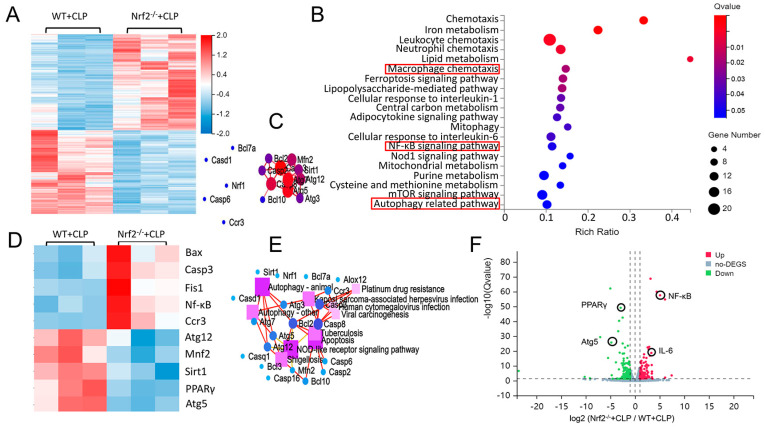
Transcriptome sequencing analyses of lung tissues in CLP-treated WT and Nrf2^−/−^ mice. (**A**) Hierarchical clustered heatmap of DEGs in lung tissues of CLP-treated WT mice (WT+CLP) and CLP-treated Nrf2^−/−^ mice (Nrf2^−/−^ + CLP); n = 6 mice per group. (**B**) KEGG enrichment analysis identified the most significantly altered signaling pathways after Nrf2 deletion; n = 3 in each group. (**C**) Construction of Nrf2-related PPI regulatory network based on DEGs. (**D**) Heatmap of DEGs in CLP-treated WT and Nrf2^−/−^ mice. (**E**) Network of KEGG pathway based on the similarity of gene expression profiles. (**F**) Volcano plot showing DEGs of CLP-treated WT and Nrf2^−/−^ mice. The red markers represent genes that were significantly upregulated, and the green markers represent genes that were significantly downregulated, while insignificantly altered genes are highlighted in gray; log2 fold change > 1, Q value < 0.05.

To analyze the mechanism underlying the biological effects of Nrf2, RNA sequencing was performed in the lung tissues of CLP-treated WT and Nrf2^−/−^ mice. In total, 3269 differentially expressed genes (log2 fold change > 1, Q value < 0.05) were created as an overall heat map. Genes that had the most significant differences (top 50) were then selected, and genes associated with autophagy phenotype were focused on and were analyzed in this manuscript. A smaller heat map was created based on these target genes, and KEGG signaling pathway analyses and protein–protein interaction analyses were performed on the genes with the most significant differences. Hierarchical clustered heatmap results indicated that after Nrf2 deletion, genes linked to apoptosis and inflammation (Bcl2, Caspase3, and NF-κB) were upregulated, and genes associated with antioxidant stress and autophagy (PPARγ, Atg5, and Atg12) were downregulated (Figure 3A,D). Kyoto Encyclopedia of Genes and Genomes (KEGG) enrichment analysis was performed to identify the most significantly altered signaling pathways after Nrf2 deletion, including the NF-κB signaling pathway and autophagy-related pathway (Figure 3B). The protein–protein interaction (PPI) regulatory network analysis predicted potential interactions among several related genes (Figure 3C). The KEGG pathway network elucidated a potential crosstalk of different signaling pathways including the autophagy and apoptosis pathways (Figure 3E). A volcano plot showed that in CLP-treated Nrf2^−/−^ mice, differentially expressed genes (DEGs) included upregulated NF-κB, and IL-6 and downregulated PPARγ and Atg5 (Figure 3F). Taken together, the RNA-seq results indicated that Nrf2 deficiency interfered with the inflammation- and autophagy-related pathways in the lung tissues of CLP mice.

### 3.4. Nrf2 Deficiency Promotes CLP-Induced Increase in M1 Macrophage Polarization and Apoptosis in Lung Tissues

**Figure 4:**
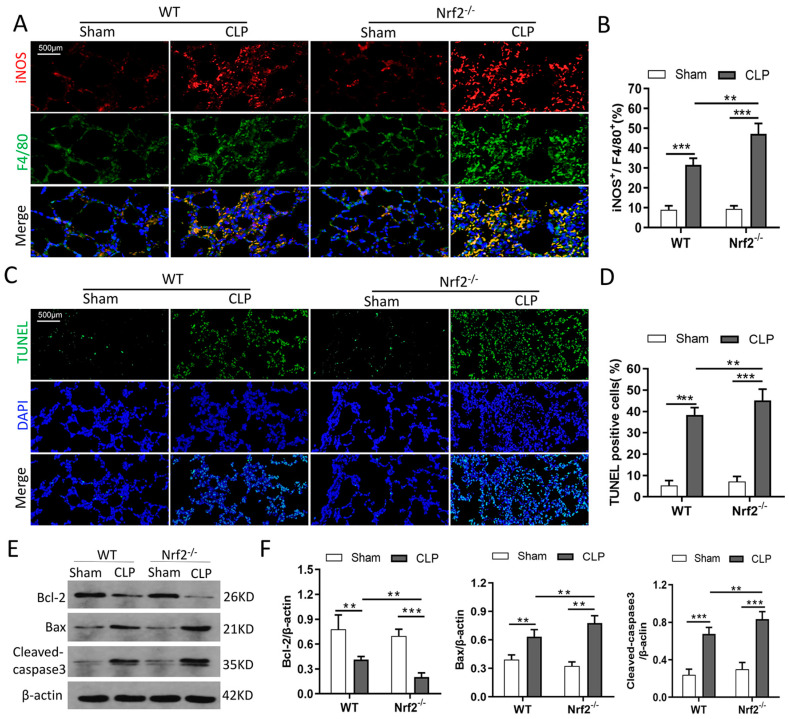
Nrf2 deficiency promotes CLP-induced M1 macrophage polarization and apoptosis in lung tissues. n = 6 mice per group. (**A**,**B**) Immunofluorescence staining of iNOS and F4/80 in the lung tissues of each group. (**C**,**D**) TUNEL staining presented apoptotic cells in the lung tissues of WT and Nrf2^−/−^ mice before or after CLP treatment. (**E**,**F**) Western blot analyses of apoptosis-associated proteins such as Bcl-2, Bax, and Cleaved—caspase3 in the lung tissues of each group. ** *p* < 0.01, *** *p* < 0.001.

The immunofluorescence staining results from pulmonary tissues showed that the expression of iNOS, an M1 macrophage marker, was significantly increased after CLP induction, and Nrf2 knockout enhanced CLP-induced increase in iNOS expression (Figure 4A,B). The TUNEL-staining results showed increased apoptosis in the lung tissues of CLP mice. Nrf2 deficiency further increased CLP-induced cell apoptosis, as reflected by the increase in TUNEL positive cells (Figure 4C,D), upregulated expressions of proapoptotic protein Bax and Cleaved-caspase3, and downregulated expression of anti-apoptotic protein Bcl-2 (Figure 4E,F). Taken together, these results indicated that Nrf2 deficiency promoted CLP-induced M1 macrophage polarization and lung tissue apoptosis.

### 3.5. Nrf2 Deficiency Inhibits CLP-Induced Upregulation of Autophagy Level in Lung Tissues

**Figure 5:**
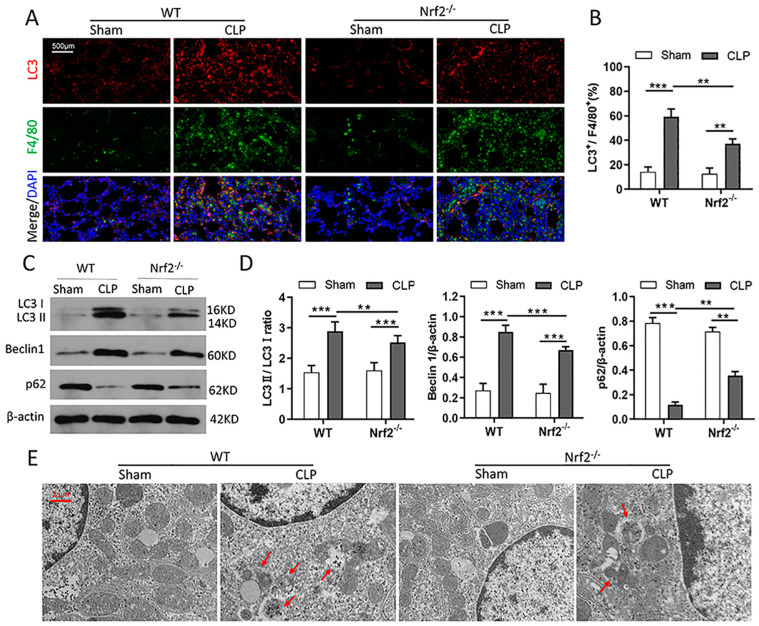
Nrf2 deficiency inhibits CLP-induced upregulation of autophagy level in lung tissues; n = 6 mice per group. (**A**,**B**) Immunofluorescence staining of LC3 and F4/80 in the lung tissues of WT and Nrf2^−/−^ mice before or after CLP treatment. (**C**,**D**) Western blot analyses of autophagy-associated proteins including LC3 I, LC3 II, Beclin1, and p62 in lung tissues of each group. ** *p* < 0.01, *** *p* < 0.001. (**E**) Transmission electron microscopy assay detected the numbers of double-membrane autophagosomes and autolysosomes (red arrows in the pictures) in lung tissues of each group.

Since several studies have reported that Nrf2 activation promotes autophagy, it was reasonable to speculate that Nrf2 deletion inhibits autophagy in CLP mice. As expected, immunofluorescence staining results showed that Nrf2 deletion alleviated the CLP-induced upregulation of the LC3 protein in pulmonary macrophages (Figure 5A,B). Autophagy-associated proteins were evaluated by Western blot, and the results showed that the LC3 II/LC3 I and Beclin1 proteins were downregulated, while the p62 protein was upregulated in Nrf2^−/−^ mice compared with WT mice after CLP treatment (Figure 5C,D). In addition, transmission electron microscopy assay was used to confirm the changes in the autophagy and autophagosome morphologies. The results showed that the numbers of autophagic vacuoles (double-membrane-bound autophagosomes) and autolysosomes (containing lysosomal membrane proteins and enzymes) were decreased in Nrf2^−/−^ mice compared with WT mice after CLP treatment (Figure 5E).

### 3.6. Nrf2 Deficiency Promotes M1 Macrophage Polarization and Inhibits M2 Macrophage Polarization through Autophagy Modulation

**Figure 6:**
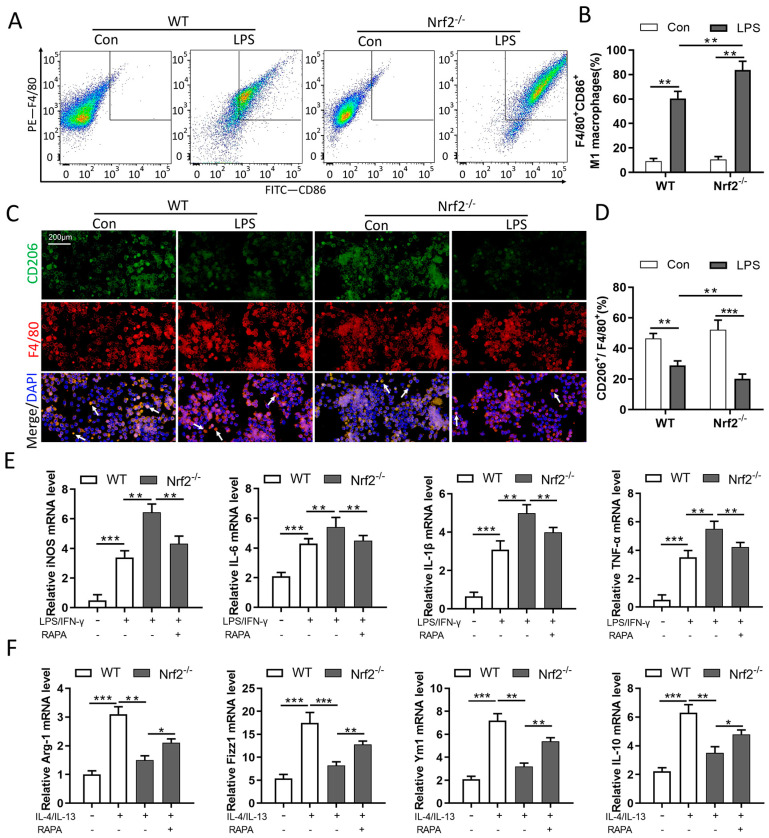
Nrf2 deficiency promotes M1 macrophage polarization and inhibits M2 macrophage polarization through autophagy modulation; n = 3 experiments per group. (**A**,**B**) Flow cytometry analyses of M1 macrophage polarization in BMDMs. (**C**,**D**) Immunofluorescence staining of CD206 and F4/80 in BMDMs. White arrows in the pictures indicate polarized macrophages. (**E**) Isolated BMDMs were treated with LPS/IFN-γ (15 ng/mL and 50 ng/mL, respectively) for 24 h to induce M1 macrophage polarization, and 0.1 μM RAPA was added simultaneously for autophagy activation. The levels of M1 macrophage markers such as iNOS, IL-6, IL-1β, and TNF-α were measured with qRT-PCR. (**F**) Isolated BMDMs were treated with IL-4/IL-13 (25 mg/mL) for 24 h to induce M2 macrophage polarization, and 0.1 μM RAPA was added simultaneously for autophagy activation. The levels of M2 macrophage markers such as Arg1, Fizz1, Ym1, and IL-10 were determined by qRT-PCR. * *p* < 0.05, ** *p* < 0.01, *** *p* < 0.001.

To verify the relationship between Nrf2 and macrophage polarization in sepsis-induced lung injury, BMDMs were isolated from WT and Nrf2^−/−^ mice and stimulated with LPS. Then, cells were collected for macrophage polarization analyses. Flow cytometry analyses showed that Nrf2 deficiency promoted an LPS-induced increase in M1 macrophage polarization (F4/80^+^/CD86^+^, as presented) (Figure 6A,B). The immunofluorescence staining results showed that Nrf2 deficiency promoted LPS-induced decrease in M2 macrophage polarization (CD206^+^/F4/80^+^, as presented) (Figure 6C,D). To further explore the involvement of autophagy in Nrf2’s regulation on macrophage polarization, LPS/IFN-γ and IL-4/IL-13 were administrated separately for the induction of M1/M2 polarization in BMDMs. The qRT-PCR analyses revealed that Nrf2 deletion exacerbated LPS/IFN-γ-induced upregulation of iNOS, IL-6, IL-1β, and TNF-α and inhibited IL-4/IL-13-induced upregulation of Arg1, Fizz1, Ym1, and IL-10 at the mRNA level. Whereas treatment with the autophagy agonist RAPA reversed the macrophage polarization phenotypes caused by Nrf2 deletion (Figure 6E,F).

### 3.7. Nrf2 Overexpression Promotes LPS-Induced Upregulation of Autophagy In Vitro

**Figure 7:**
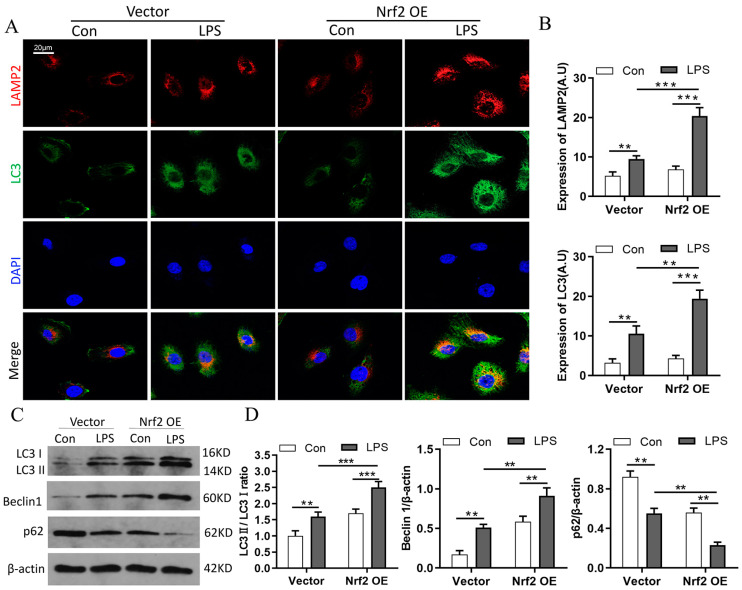
Nrf2 overexpression promotes LPS-induced upregulation of autophagy in vitro; n = 3 experiments per group. (**A**,**B**) Immunofluorescence staining of LAMP2 and LC3 in RAW264.7 cells. (**C**,**D**) Western blot analyses of the levels of LC3 I, LC3 II, Beclin1, and p62 proteins in RAW264.7 cells of each group. ** *p* < 0.01, *** *p* < 0.001.

The relationship between Nrf2 and autophagy was further examined in vitro in RAW264.7 cells transfected with empty vector or Nrf2 plasmid. Immunofluorescence staining results showed that Nrf2 overexpression promoted the expressions as well as the co-localization of LAMP2 and LC3 proteins in RAW264.7 cells with LPS treatment (Figure 7A,B). Furthermore, Western blot analyses revealed that Nrf2 overexpression promoted LPS-induced upregulation of LC3 II/LC3 I and Beclin1 and downregulation of p62 (Figure 7C,D).

### 3.8. Nrf2 Overexpression Inhibits M1 Macrophage Polarization and Improves M2 Macrophage Polarization by Promoting Autophagy In Vitro

**Figure 8:**
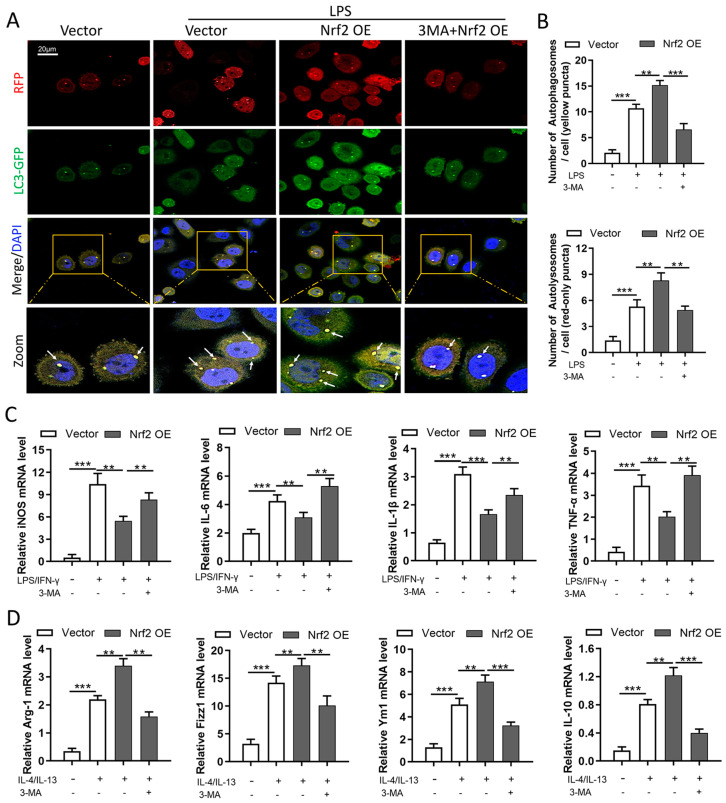
Nrf2 overexpression inhibits M1 macrophage polarization and improves M2 macrophage polarization by promoting autophagy in vitro. Macrophage polarization induction was performed in RAW264.7 cells; n = 3 experiments per group. (**A**,**B**) Cells were first transfected with 2 μg of Nrf2 plasmid or vector for 48 h and treated with 1 μg/mL LPS for 24 h. Then, mRFP-GFP-LC3B plasmids were transfected, and 24 h later confocal images were taken to detect the numbers of autophagosomes (yellow dots) and autolysosomes (red dots). White arrows indicate autophagosomes in merged images. (**C**) RAW264.7 cells were treated with LPS/IFN-γ (15 ng/mL and 50 ng/mL, respectively) for 24 h to induce M1 macrophage polarization, and 2 mM 3-MA was added simultaneously for autophagy inhibition. The levels of M1 macrophage markers including iNOS, IL-6, IL-1β, and TNF-α were measured with qRT-PCR. (**D**) RAW264.7 cells were treated with IL-4/IL-13 (25 mg/mL) for 24 h to induce M2 macrophage polarization, and 2 mM 3-MA was added simultaneously for autophagy inhibition. The levels of M2 macrophage markers including Arg1, Fizz1, Ym1, and IL-10 were measured with qRT-PCR. ** *p* < 0.01, *** *p* < 0.001.

We next determined autophagic flux using the mRFP-GFP-LC3B dual-fluorescence autophagy-detection plasmid. In this system, a reduction in the green GFP fluorescence indicates that lysosomes fuse with autophagosomes to form autolysosomes [27,28]. Confocal images revealed that Nrf2 overexpression promoted an LPS-induced increase in autophagosomes and autolysosomes, and 3-MA treatment significantly inhibited the formation of autophagosomes and autolysosomes (Figure 8A,B). In addition, Nrf2 overexpression inhibited LPS/IFN-γ-induced upregulation of iNOS, IL-6, IL-1β, and TNF-α and promoted IL-4/IL-13-induced upregulation of Arg1, Fizz1, Ym1, and IL-10 at the mRNA level, whereas treatment with 3-MA partially reversed the macrophage polarization phenotype caused by Nrf2 overexpression (Figure 8C,D).

### 3.9. Nrf2 Overexpression In Vitro Promotes PPARγ but Inhibits NF-κB Nuclear Translocation

**Figure 9:**
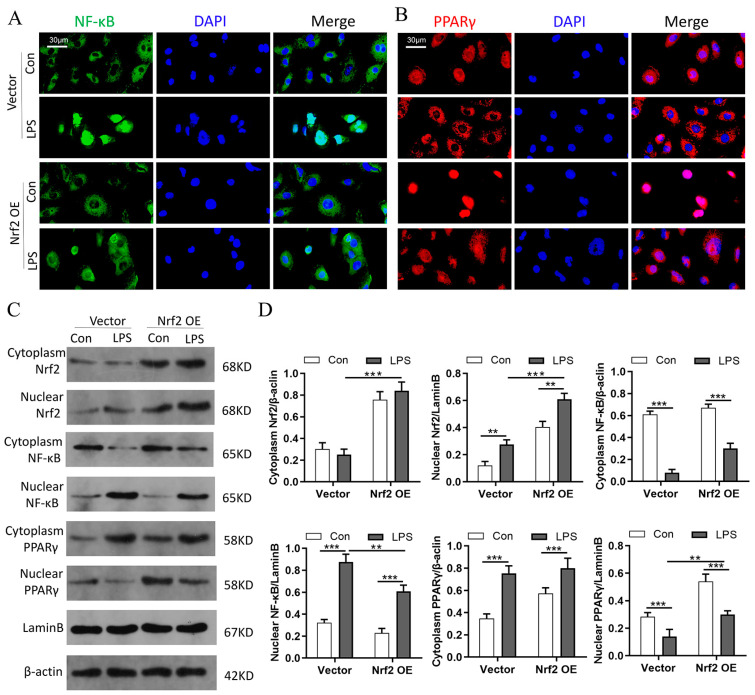
Nrf2 overexpression in vitro promotes PPARγ but inhibits NF-κB nuclear translocation; n = 3 experiments per group. (**A**,**B**) Immunofluorescence staining of NF-κB and PPARγ in RAW264.7 cells in each group. (**C**,**D**) Western blot analyses of Nrf2, NF-κB, and PPARγ levels in both cytoplasm and nucleus. ** *p* < 0.01, *** *p* < 0.001.

Macrophage polarization was regulated by transcription factors. Increasing evidence has revealed that NF-κB is associated with M1 macrophage activation, while PPARγ could regulate M2 macrophage polarization [29,30]. Moreover, PPARγ activation has been shown to reverse M1/M2 macrophage polarization through direct interaction with NF-κB [30]. Therefore, it was reasonable to speculate that Nrf2 regulated macrophage polarization through the NF-κB/PPARγ signaling pathways. The immunofluorescence staining results in RAW264.7 cells showed that Nrf2 overexpression inhibited LPS-induced NF-κB entry into the nucleus and promoted PPARγ nuclear translocation (Figure 9A,B). Western blot analyses showed that Nrf2 overexpression inhibited LPS-induced upregulation of NF-κB in the nucleus and significantly promoted PPARγ expression in the nucleus (Figure 9C,D). Taken together, these results suggested that the regulatory effect of Nrf2 on macrophage polarization is at least partially realized through the inhibition of NF-κB signaling and activation of PPARγ pathway.

## 4. Discussion

In the present study, clinical samples from patients with sepsis were collected and analyzed, and a CLP-treated Nrf2-knockout mouse model was established to investigate the role of Nrf2 involved in sepsis-induced ALI. Our results suggested that Nrf2 deficiency exacerbated sepsis-induced lung injury and inflammation. Clinical studies indicated that in patients with sepsis, NRF2 expression was negatively correlated with disease severity and lung inflammation. In vivo experiments revealed that Nrf2 deficiency promoted M1 macrophage polarization and inhibited M2 macrophage polarization through the inhibition of macrophage autophagy. In RAW264.7 cells exposed to LPS, Nrf2 overexpression inhibited M1 macrophage polarization and promoted M2 macrophage polarization through improving autophagy. Furthermore, in vitro experiments showed that Nrf2 overexpression promoted PPARγ expression in the nucleus and inhibited LPS-induced NF-κB nuclear translocation. These results indicate that Nrf2 deficiency promotes sepsis-induced pulmonary injury through the regulation of autophagy- and NF-κB/PPARγ-mediated macrophage polarization.

NRF2 plays an important protective role in a series of lung diseases such as pneumonia, asthma, emphysema, ALI, and pulmonary fibrosis [31,32,33,34,35]. As an important regulator of redox homeostasis, NRF2 is involved in the regulation of more than 500 genes, including genes that participate in oxidative stress (HO-1, TXNRD1), inflammation (TGF-β, NF-κB), apoptosis (Bcl-2, BclxL), autophagy (p62), and cellular bioenergetics (G6PD, PPARγ) [35,36,37]. In the present study, we mainly focused on the regulatory effect of Nrf2 on inflammatory response in sepsis-induced ALI. It has been reported that the Nrf2 pathway is involved in the suppression of LPS-induced lung inflammation [38,39]. In vivo and in vitro experiments showed that Honokiol inhibited NLRP3-inflammasome-mediated pyroptosis through Nrf2 activation, thereby alleviating LPS-induced ALI [5]. In a recent study of terretonin, a meroterpenoid against LPS-induced ALI, researchers revealed that LPS induced the downregulation of Nrf2 expression as well as its binding activity [40]. By using siRNA-mediated Nrf2-knockdown and Nrf2-activation methods, researchers found that Nrf2 inhibited LPS-induced inflammation by promoting polarization of M2 macrophages and inhibiting polarization of M1 macrophages, which is similar to our results [11].

The relationship between autophagy and inflammation in pulmonary disease has been focused on in previous studies [41]. Autophagy in alveolar macrophages was reported to play an important role in inhibiting spontaneous pulmonary inflammatory responses [42]. An autophagy-related proteins-associated autophagy deficiency intensified neutrophilic inflammation and resulted in severe lung injury in mice [43,44]. These results suggest that autophagy plays a protective role in the host defense response during acute pulmonary infection. However, excessive autophagy could produce adverse effects and lead to ALI in the late stage of inflammation [45], and an excess of autophagosomes could transform a normal cellular protection response into a harmful stimulus [46,47]. In patients with chronic obstructive pulmonary disease (COPD) who smoke, the role of autophagy involved in the pathogenesis of pulmonary inflammation also remains controversial. Some identified autophagy activation as a detrimental effect in lung epithelial cells response to smoke [48,49,50], while others reported that reduced autophagic flux in smokers’ alveolar macrophages resulted in defective delivery of bacteria to lysosomes and an autophagy/lysosomal functional deficiency [51]. These results indicate that the effect of autophagy in pulmonary inflammatory disease is related to the development stage of the disease and the type of cell that produces autophagy. In the present study, Nrf2 deficiency promoted M1 macrophage polarization and inflammation via inhibition of autophagy in mouse BMDMs. In others’ research, Nrf2 deletion significantly increased ischemia/reperfusion-induced upregulation of autophagosomes and autophagy-related proteins, as well as the proinflammatory factors [21]. As for the effect of Nrf2 on autophagy in inflammation, our conclusion is different from others. A possible explanation is that we focused on autophagy in macrophages, while others carried out autophagy research on lung epithelial cells. In addition, it was reported that Nrf2 activation in the early “cytokine storm” phase of acute inflammation suppressed LPS-induced lung inflammation [11]. In the present study, LPS-induced autophagy effects reached a maximum at 24 h, which was also the time point for drug administration and inflammation detection, and, with the further extension of LPS treatment time, the numbers of autophagosomes and autolysosomes decreased (see Supplementary Materials). These results indicate that in an LPS-induced injury model, the effects of autophagy and inflammation are related to the duration of injury, and the inflammatory protective effects of Nrf2 may occur in the early stage of injury.

Accumulating evidence has revealed the relationship between autophagy and macrophage polarization in inflammatory diseases [52]. For example, anti-inflammatory plant Araloside C (AsC) was reported to polarize macrophages towards M2 phenotype and enhance oxidative low-density lipoprotein-induced macrophage autophagy. The inhibition of autophagy could abolish the anti-atherosclerosis and M2 macrophage polarization effects of AsC [53]. As a histone deacetylase inhibitor, Trichostatin A was indicated to increase the M2 macrophage phenotype and reduce inflammation by enhancing autophagy during polymicrobial sepsis [54]. In addition, with the knockout of Atg5 in the macrophages of high-fat-diet-fed LPS-stimulated mice, researchers found that the loss of autophagic function in macrophages led to a proinflammatory state [55]. However, the regulatory effects of Nrf2 on both autophagy and macrophage polarization, as well as the mechanisms by which Nrf2 regulates autophagy and macrophage polarization to exert an anti-inflammatory effect, remain unclear. In the present study, we observed that Nrf2 deficiency resulted in decreased autophagy-mediated macrophage polarization from M2 to M1 and finally exacerbated sepsis-induced pulmonary injury and inflammation. These findings may well explain the anti-inflammatory role of Nrf2 in autophagy-mediated macrophage polarization, so it is reasonable to suggest that targeting Nrf2 might provide a potential treatment for sepsis-induced pulmonary inflammatory injury.

In the present study, the results revealed that Nrf2 overexpression promoted the polarization of macrophages into the M2 phenotype, and, in LPS-treated RAW264.7 cells, Nrf2 overexpression inhibited NF-κB but promoted nuclear PPARγ expression, which was similar to the results of previous studies [11]. In fact, the effects of modulation of the NF-κB or PPARγ pathway on M1/M2 macrophage polarization have been reported in many studies [29,56,57,58]. In non-alcoholic fatty liver disease, upregulation of PPARγ expression significantly inhibited saturated fatty acids-induced NF-κB activation and resulted in macrophage polarization into an M2 phenotype [30]. In a methicillin-resistant staphylococcus aureus-induced murine ALI model, the anti-inflammatory substance dehydrocostus lactone induced polarization of the macrophages from M1 to M2 by inhibiting the p38 MAPK/NF-κB signal and activating the Nrf2 signal [12]. Therefore, we conclude that Nrf2 regulates macrophage polarization through interference of the NF-κB and PPARγ signaling pathways. In addition, several studies revealed that autophagy was involved in macrophage inflammatory response via the NF-κB/PPARγ signaling pathways. For example, in non-alcoholic steatohepatitis (NASH), HIF-1α activation and decreased autophagic flux stimulated inflammation in macrophages by upregulation of the NF-κB pathway [59]. Atg5-deficiency-mediated mitophagy aggravated cardiac inflammation by activating NF-κB in macrophages [60]. In adipose tissue macrophages, palmitate internalization was reported to activate p62 and PPARγ, thereby promoting lipid metabolism and limiting inflammation [61]. In post-hemorrhagic shock acute lung inflammation, autophagy induction in the alveolar macrophage decreased inflammatory cytokines expression by inhibiting NF-κB pathway [62]. Based on the above findings, we draw the conclusion that an Nrf2 deficiency exacerbates CLP-induced pulmonary injury and inflammation through autophagy-and NF-κB/PPARγ-mediated macrophage polarization.

## 5. Conclusions

**Figure 10:**
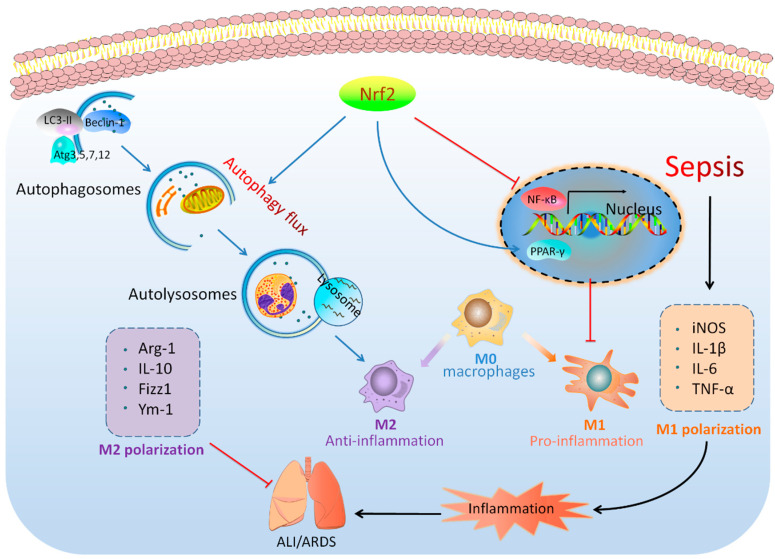
Hypothetical schema for the roles of Nrf2 in sepsis-induced lung injury. Nrf2 plays a protective role in sepsis-induced lung injury and inflammation through the regulation of autophagy-and NF-κB/PPARγ-mediated macrophage polarization.

In conclusion, our clinical studies, as well as the in vivo and in vitro experiments, show that Nrf2 plays a protective role in sepsis-induced lung injury and inflammation by the regulation of autophagy-and NF-κB/PPARγ-mediated macrophage polarization. The hypothetical schema for the roles of Nrf2 in sepsis-induced lung injury are presented in Figure 10. Therefore, Nrf2 could be considered as a potential therapeutic target for sepsis-induced lung injury.

## Figures and Tables

**Table 1:** Demographic data, comorbidities, and laboratory test results of healthy controls and sepsis patients.

Variables	Healthy Controls (n = 30)	Sepsis Patients (Acute Stage, n = 30)	*p* Value
Age (years; mean ± SD)	56.23 ± 11.23	54.58 ± 13.87	0.615 ^a^
Male gender (n, %)	21 (70.00)	23 (76.67)	0.559 ^b^
Comorbidities (n, %)			
Hypertension	7 (23.33)	10 (33.33)	0.390 ^b^
Coronary heart diseases	6 (20.00)	3 (10.00)	0.608 ^b^
Diabetes	2 (0.07)	5 (0.17)	0.421 ^b^
Complete blood count (mean ± SD or median (interquartile range))			
WBC (×10^9^/L)	8.31 ± 4.61	11.78 ± 7.49	**0.034** ^a^
LYM (×10^9^/L)	0.51 (0.32–0.91)	0.42 (0.44–0.79)	0.331 ^c^
NEU (×10^9^/L)	5.39 (3.81–6.73)	13.51 (7.11–18.78)	**0.017** ^c^
MONO (×10^9^/L)	0.39 ± 0.33	0.50 ± 0.38	0.236 ^a^
HGB (g/L)	112.32 ± 21.87	105.57 ± 26.58	0.287 ^a^
PLT (×10^9^/L)	150.73 ± 83.45	145.28 ± 90.92	0.809 ^a^
Blood gas analysis (mean ± SD)			
SaO_2_	0.98 ± 0.02	0.87 ± 0.23	**0.012** ^a^
PCO_2_ (mmHg)	35.65 ± 8.45	58.53 ± 15.37	**<0.001** ^a^
PO_2_ (mmHg)	146.91 ± 56.34	72.35 ± 35.34	**<0.001** ^a^
PH	7.38 ± 0.12	7.24 ± 0.23	**0.004** ^a^
BE (mmol/L)	−1.29 ± 1.88	−5.47 ± 6.70	**0.003** ^a^
HCO_3_^−^ (mmol/L)	18.26 ± 4.33	17.57 ± 8.13	0.683 ^a^
Lac (mmol/L)	1.81 ± 0.85	2.77 ± 1.92	**0.017** ^a^
Inflammatory profile (median (interquartile range))			
PCT (ng/mL)	0.72 (0.21–2.83)	3.58 (2.45–8.39)	**0.007** ^c^
CRP (mg/L)	82.65 (23.45–130.39)	145.77 (56.21–312.40)	**0.008** ^c^
Biochemical test (mean ± SD or median (interquartile range))			
TP (g/L)	65.38 ± 8.71	62.67 ± 6.34	0.174 ^a^
GLB (g/L)	31.45 ± 7.32	30.22 ± 8.19	0.542 ^a^
ALB (g/L)	35.73 ± 5.75	33.30 ± 6.36	0.126 ^a^
ALT (U/L)	38.41 ± 12.15	40.33 ± 9.16	0.492 ^a^
AST (U/L)	55.97 ± 6.31	53.53 ± 7.52	0.807 ^a^
BUN (mmol/L)	9.32 ± 3.75	10.54 ± 2.33	0.136 ^a^
Cr (μmol/L)	94.22 ± 15.76	101.75 ± 22.97	0.144 ^a^
UA (μmol/L)	306.19 ± 98.23	345.74 ± 112.13	0.152 ^a^
LDH (U/L)	152.42 ± 34.16	157.83 ± 22.74	0.473 ^a^
BNP (pg/mL)	126.21 ± 32.67	143.37 ± 26.75	**0.030** ^a^
CK-MB (U/L)	30.51 (11.01–23.26)	31.87 (15.71–44.61)	0.271 ^c^
cTnI (pg/mL)	0.01 (0.00–0.13)	0.01 (0.00–0.21)	0.890 ^c^
K^+^ (mmol/L)	4.31 ± 0.75	4.27 ± 0.81	0.843 ^a^
Na^+^ (mmol/L)	137.23 ± 15.21	134.56 ± 14.23	0.485 ^a^

Abbreviations: WBC, white blood cell; LYM, lymphocyte; NEU, neutrophil; MONO, monocyte; HGB, hemoglobin; PLT, platelet; PGT, procalcitonin; CRP, C-reactive protein; TP, total plasma protein; GLB, globulin; ALB, albumin; ALT, alanine aminotransferase; AST, aspartate aminotransferase; BUN, blood urine nitrogen; Cr, creatinine; UA, uric acid; LDH, lactate dehydrogenase; BNP, brain natriuretic peptide; CK-MB, creatinine kinase-MB; cTnI, highly sensitive troponin I. ^a^ Student’s t-test; ^b^ χ^2^ test; ^c^ Mann–Whitney *U* test.

## Data Availability

The original contributions presented in the study are included in the article/Supplementary Materials. The datasets generated for this study can be found in NCBI (https://dataview.ncbi.nlm.nih.gov/object/PRJNA786443?reviewer=17j2pks6qk33ltt2m7tnhv9cdc) with the BioProject ID: PRJNA786443.

## Supplementary Materials

The following supporting information can be downloaded at: https://www.mdpi.com/article/10.3390/cells11233927/s1. Table S1: Primers used in the study. Figure S1: The transfection efficiency of Nrf2 plasmid was detected by qRT-PCR and Western blot. Figure S2: The vector maps used in the study. Figure S3: Preliminary experiments on the concentration and timing selection of LPS treatment in in vitro experiments.
