# Supplementary material for: Nrf2 Deficiency Exacerbated CLP-Induced Pulmonary Injury and Inflammation through Autophagy- and NF-κB/PPARγ-Mediated Macrophage Polarization
Source: Cells. 2022 Dec 4;11(23):3927. doi: 10.3390/cells11233927 (PMC9735993; doi:10.3390/cells11233927)
Supplement: Supplementary file 1 [file cells-11-03927-s001.zip › cells-2032138-supplementary.pdf]

Supplementary Information

Nrf2 Deficiency Exacerbated CLP-induced Pulmonary Injury and Inflammation through Autophagy- and NF-κB/PPARγ- Mediated Macrophage Polarization

Jing Luo[1,†], Jin Wang[1,†], Jing Zhang[1], Aming Sang[1], Xujun Ye[2], Zhenshun Cheng[3,4,5,*], Xinyi Li[1,*]

[1] Department of Anesthesiology, Zhongnan Hospital of Wuhan University, Wuhan 430071, China

[2] Department of Geriatrics, Zhongnan Hospital of Wuhan University, Wuhan 430071, China

[3] Department of Respiratory and Critical Care Medicine, Zhongnan Hospital of Wuhan University, Wuhan 430071, China

[4] Wuhan Research Center for Infectious Diseases and Cancer, Chinese Academy of Medical Sciences, Wuhan 430071, China

[5] Hubei Engineering Center for Infectious Disease Prevention, Control and Treatment, Wuhan 430071, China

[*]Correspondence: chzs1990@163.com (Z.C.); lxy08272021@126.com or zn000484@whu.edu.cn (X.L.)

[†] These authors contributed equally to this work.

Supplementary Table S1: Primers used in the study.

Gene	Forward primer (5'-3')	Reverse primer (5'-3')
Homo NRF2	AGGTTGCCCACATTCCCAAA	AGTGACTGAAACGTAGCCGA
Homo ACTB	GCACAGAGCCTCGCCTT	GTTGTCGACGACGAGCG
Mus Nrf2	CTTTAGTCAGCGACAGAAGGAC	AGGCATCTTGTTTGGGAATGTG
Mus iNOS	GAGCAACTACTGCTGGTGGT	CGATGTCATGAGCAAAGGCG
Mus IL-6	TGTATGAACAACGATGATGCACTT	ACTCTGGCTTTGTCTTTCTTGTTATCT
Mus IL-1β	CTGGTACATCAGCACCTCAC	AGAAACAGTCCAGCCCATAC
Mus TNF-α	AGTGACAAGCCTGTAGCCC	GAGGTTGACTTTCTCCTGGTAT
Mus Arg-1	ACATTGGCTTGCGAGACGTA	TCCATCACCTTGCCAATCCC
Mus Fizz1	GGGATGACTGCTACTGGGTG	TCAACGAGTAAGCACAGGCA
Mus Ym1	TCCTACTGGAAGGACCATGGAGCA	TCCTGGTGGGCCAGTACTAATTGT
Mus IL-10	GCTCTTACTGACTGGCATGAG	CGCAGCTCTAGGAGCATGTG
Mus β-actin	AGTGTGACGTTGACATCCGT	GCAGCTCAGTAACAGTCCGC

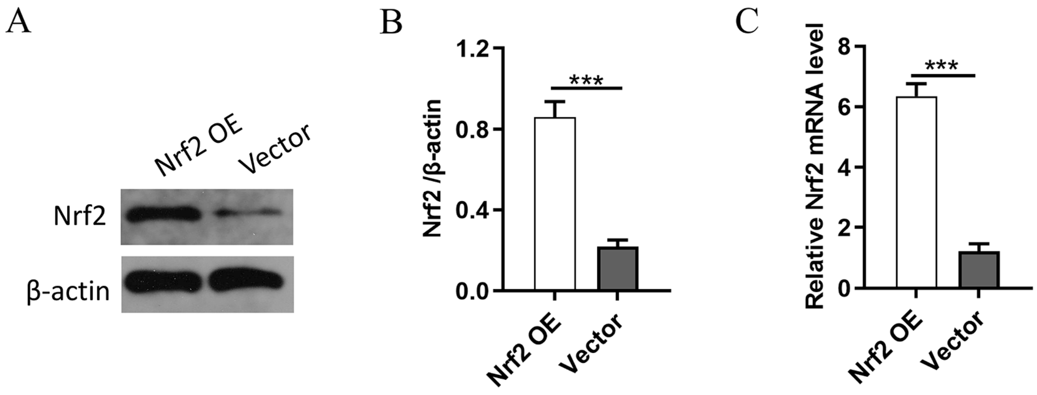

Supplementary Figure S1. The transfection efficiency of Nrf2 plasmid was detected by qRT-PCR and western blot. (**A**, **B**) Western blot analysis of Nrf2 protein in Nrf2 overexpression group and the control group. (**C**) The expression of Nrf2 mRNA level was measured with qRT-PCR in each group. ***$p < 0.001$.

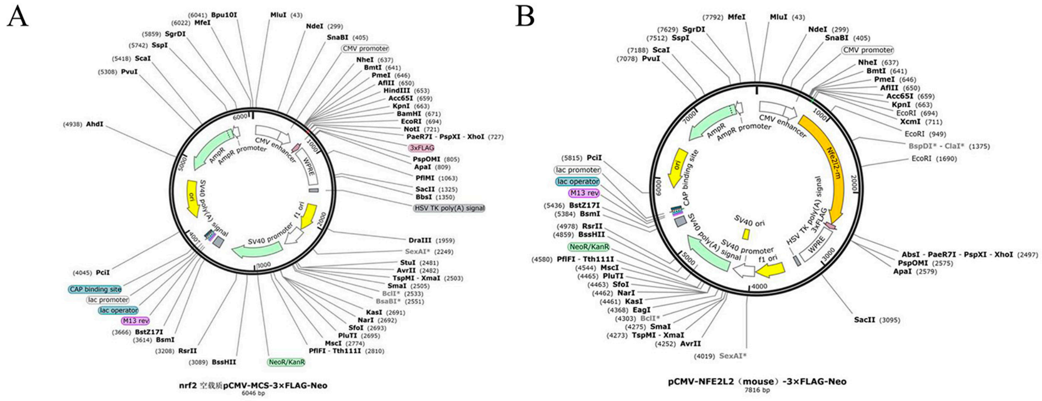

Supplementary Figure S2. (**A**) The vector map of the "Vector" group. (**B**) The vector map of the "Nrf2 OE" group. The primer sequences for amplifying Nrf2 were as follows: Forward primer (5'-3') AGTCCAGTGTGGTGGAATTCGCCACCatgatggacttggagttgccac, Reverse primer (5'-3') TGGTCTTTGTAGTCCTCGAGgtttttctttgtatctggcttcttg.

To construct LPS-induced inflammation model in RAW264.7 cells, we explored the appropriate concentration and treatment time of LPS. Firstly, RAW264.7 cells were treated with different concentrations of LPS (0.5 μg/mL, 1 μg/mL, 2 μg/mL, 5 μg/mL) for 24 h. The CCK-8 kit (Sigma-Aldrich, Saint Louis, MO, USA, 96992) was used for the quantitation of viable cell number. Apoptotic cells were labeled with Annexin V (Sigma-Aldrich, Saint Louis, MO, USA, APOAF-20TST) and analyzed by flow cytometry. Results showed that compared with the control group, the number of cells was significantly reduced and the proportion of apoptotic cells was significantly increased when LPS concentration was greater than 1 μg/mL, so 1 μg/mL was chosen as the appropriate concentration of LPS. Secondly, RAW264.7 cells were treated with 1 μg/mL LPS for 6 h, 12 h, 24 h and 48 h, and the number of cells was detected and the apoptosis was analyzed. The results indicated that the number of cells was significantly decreased compared with the undamaged control group when the treatment time of 1 μg/mL LPS was 12 h or longer. Flow

cytometry results showed that the proportion of apoptotic cells increased significantly when the treatment time of 1 μg/mL LPS was 6 h or longer. We then detected the number of autophagosomes and autolysosomes using the mRFP-GFP-LC3B plasmid, a reporting system that reflects autophagic flux. The results revealed that 1 μg/mL LPS-induced autophagy effects reached a maximum at 24 h, and with the prolongation of LPS treatment time, the numbers of autophagosomes and autolysosomes decreased significantly. Based on the above results, 24 h was chosen as the optimum treatment time of LPS.

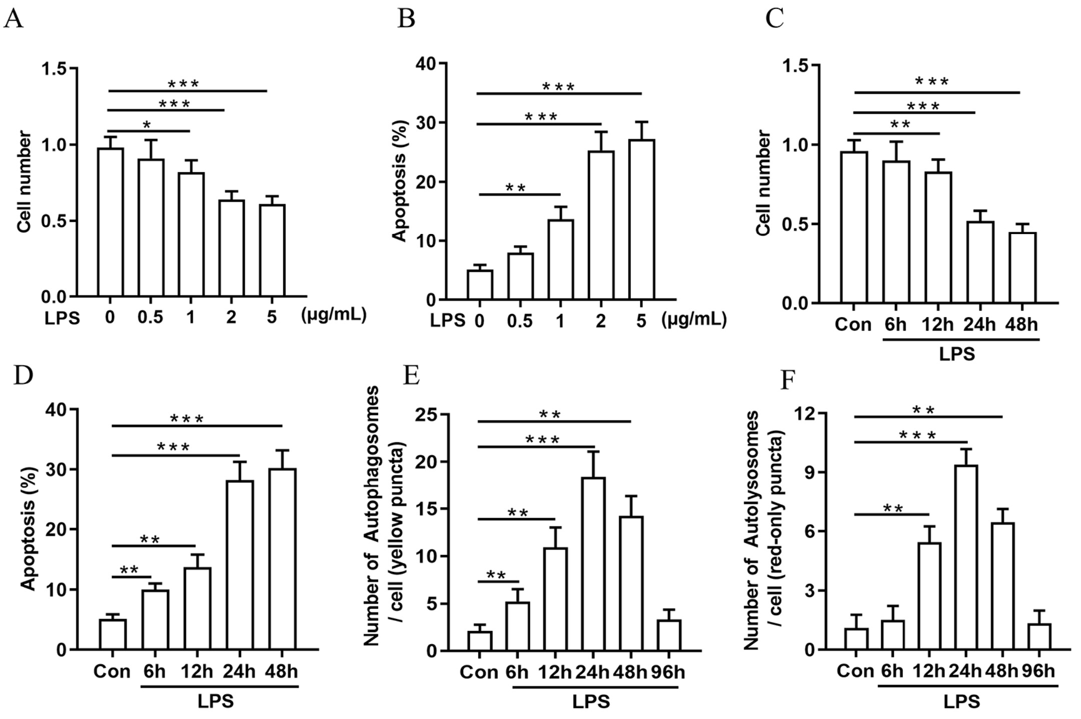

Supplementary Figure S3. Preliminary experiments on the concentration and timing selection of LPS treatment in in vitro experiments. (**A**) RAW264.7 cells were treated with LPS at different concentrations (0.5 μg/mL, 1 μg/mL, 2 μg/mL, 5 μg/mL) for 24 h, and the number of living cells was detected by CCK-8 kit. (**B**) RAW264.7 cells were treated with LPS at different concentrations (0.5 μg/mL, 1 μg/mL, 2 μg/mL, 5 μg/mL) for 24 h, and the percentage of apoptotic cells was detected by flow cytometry. (**C**) RAW264.7 cells were treated with 1 μg/mL LPS for different time (6 h, 12 h, 24 h, 48 h), and the number of living cells was detected by CCK-8 kit. (**D**) RAW264.7 cells were treated with 1 μg/mL LPS for different time (6 h, 12 h, 24 h, 48 h), and the percentage of apoptotic cells was detected by flow cytometry. (**E**, **F**) Cells were treated with 1 μg/mL LPS for different time (6 h, 12 h, 24 h, 48 h, 96 h), and then transfected with the mRFP-GFP-LC3B plasmid, 24 h later confocal images were taken to detect the numbers of autophagosomes and autolysosomes. *$p < 0.05$, **$p < 0.01$, ***$p < 0.001$.
